# How we treat diarrhea in pediatric transplant patients: a brief review

**DOI:** 10.3389/fped.2023.1287445

**Published:** 2023-12-08

**Authors:** Timothy Dean Minniear, Surabhi Vora

**Affiliations:** ^1^ Division of Pediatric Infectious Diseases, University of Tennessee Health Science Center, Memphis, TN, United States; ^2^ Division of Infectious Diseases, Seattle Children’s Hospital, Seattle, WA, United States; ^3^ Department of Pediatric Infectious Diseases, University of Washington, Seattle, WA, United States

**Keywords:** infectious diarrhea, solid organ transplant (SOT), haematopoetic stem cell therapy, bone marrow translation (BMT), clostridium diffcile colitis, CMV colitis, bacterial diarrhea in children, viral diarrhea

## Abstract

Diarrhea is a common problem faced by both hematopoietic and solid organ transplant recipients. The differential diagnosis is wide, ranging from infectious to non-infectious causes and from benign to emergent illness. Here we present two patients with diarrhea and discuss our approaches to the diagnostic evaluation and management of transplant recipients with diarrhea. We also include a review of the literature and discuss areas in need of further study.

## Background

Diarrhea is frequently observed and can cause significant morbidity in pediatric transplant patients, both in solid organ transplant (SOT) (1, 2) and hematopoietic cell transplant (HCT) settings. The differential is broad and often multifactorial and therefore diagnosis can be complicated. Supportive care is the mainstay of treatment but reduction of immune suppression and antimicrobial therapy may be appropriate in some cases, depending on the etiology.

In this review we describe two cases of pediatric transplant patients who present with diarrhea, discuss the differential diagnosis and outline a practical approach to diagnosis and management.

## Case 1

An eight-year-old male with a history of acute lymphoblastic leukemia (ALL) now 128 days following matched peripheral blood stem cell transplant presented to the emergency department (ED) with one week of nausea, decreased appetite and acute diarrhea. His parents described up to three episodes of loose to watery stools each day without blood. He had a fever of 103 degrees fahrenheit on the day of presentation. Review of symptoms also revealed upper respiratory symptoms including cough and runny nose. He has not had significant abdominal pain, new rashes, dysuria or episodes of vomiting. Infectious history includes pulmonary nodules on imaging that largely resolved by 100 days following hematopoietic cell transplantation (HCT) at which time empiric antifungal treatment was discontinued. The patient also had a history of *Clostridium difficile* colitis one month after HCT (approximately 3 months prior to current presentation) which was treated with 10 days of oral vancomycin. He remained on antimicrobial prophylaxis with acyclovir and trimethoprim-sulfamethoxazole. The patient also had a history of grade IIa gut graft vs. host disease (GVHD), which was treated with prednisolone and budesonide until one month prior to his current presentation. He remained on tacrolimus for continued GVHD prophylaxis. He also had a history of hypogammaglobulinemia with the most recent IgG level one month prior of 531 mg/dl.

Upon presentation to the ED he was afebrile with normal vital signs. On exam, he had rhinorrhea, mild diffuse abdominal tenderness without rebound, guarding or masses. The rest of his exam was unremarkable. Laboratory evaluation was significant for an alanine aminotransferase of 68 U/L and aspartate aminotransferase of 81 U/L, which are both stable from prior assessments. The white blood count was 5.2 K/mm^3^ with an absolute neutrophil count of 3,200/mm^3^ and absolute lymphocyte count of 750/mm^3^. Serum viral polymerase chain reaction (PCR) tests one week prior to the current presentation were all negative including cytomegalovirus (CMV), Epstein-Barr virus (EBV) and Adenovirus (ADV). He received no recent antibiotics apart from the antimicrobial prophylaxis already mentioned.

Given his new respiratory symptoms, a rapid respiratory viral PCR panel (BioFire FilmArray) was performed and resulted positive for rhinovirus/enterovirus as well as ADV. Stool studies were ordered including *Clostridium difficile* toxin assay, ADV specific PCR and a multiplex PCR panel (BioFire FilmArray Gastrointestinal Panel), all of which came back negative. Serum ADV PCR was also obtained and resulted with 12,589 copies/ml (log 4.1). A quantitative respiratory viral PCR test (University of Washington) demonstrated an adenovirus CT value of 31 and a rhinovirus CT 25. Blood cultures remained negative. Chest x-ray was obtained and was consistent with viral respiratory infection.

Over the next few days his fever and diarrhea resolved and ADV level in the blood trended down. Treatment consisted of supportive care alone without antimicrobial therapy.

## Case 2

A 21-month-old, CMV donor negative/recipient negative, EBV donor positive/recipient negative female heart transplant recipient was admitted for fever, vomiting and diarrhea 208 days after transplant. On exam, she was fussy but consolable, well hydrated, and had a single 1 cm × 0.5 cm shallow ulceration on the tip of her tongue. Stools were mucoid and non-bloody at presentation. She was immunosuppressed with tacrolimus and sirolimus without an antimetabolite. She was also on amoxicillin prophylaxis for asplenia.

Past infectious history included primary CMV infection 33 days after transplant and was treated with valganciclovir and had been undetectable at presentation. She had also developed fever and exudative pharyngitis with associated EBV viremia 138 days after transplant. Symptoms resolved promptly and EBV viral load remained stable at ∼3.25 log copies/ml.

A stool sample for qualitative multiplex PCR (BioFire FilmArray Gastrointestinal Panel) collected on admission was negative. Serum CMV viral load was <2.14 log IU/ml and EBV viral load was 4.39 log copies/ml. Gastroenterology was consulted for colonoscopy and, due to the tongue ulceration, endoscopy. There was a small mucosal ulcer in the distal esophagus but otherwise the endoscopy was normal. The colonoscopy demonstrated multiple shallow and deep ulcerations with mounded edges and no active bleeding. Biopsies confirmed post-transplant lymphoproliferative disease (PTLD) in the descending and sigmoid colon with positive CD20 and EBER stains. She was referred to oncology for PET scan, which revealed diffuse intestinal involvement and three cervical sites, and received treatment with cyclophosphamide, hydroxydaunorubicin, vincristine, and prednisone (CHOP) following which her PET scan showed resolution of lesions and colonoscopy was negative for lesions by 77 days after diagnosis.

## Differential diagnosis

Diarrhea in the immunocompromised host can stem from a variety of causes and can certainly be multifactorial (Table 1). Medication toxicity is common due to the variety of chemotherapeutic agents, conditioning agents, GVHD preventative and treatment medications, nutritional supplements and other therapies these patients receive. Malabsorption and mucositis frequently affect transplant recipients prior to the transplant itself as well as potentially for a period afterwards. Antibiotics themselves can disrupt the gut microbiome and cause transient or longer lasting diarrhea. In HCT patients, GVHD which affects the gut can cause mild to severe diarrhea. On the other hand, PTLD is more likely to impact SOT patients. Finally, infectious causes of diarrhea in transplant patients are many and will be discussed in further detail below. Most commonly, these include viral causes, bacterial infections including *Clostridium difficile* and parasitic organisms.

**Table 1:** Differential diagnosis of diarrhea in pediatric transplant patients (non-exhaustive).

Infectious Causes	Type	Pathogens	Common Diagnostic Tools
	Bacterial	*Campylobacter* *Clostridium difficile* *Escherichia coli* *Salmonella* *Shigella* *Vibrio* *Yersinia*	Multiplex stool PCR panel Stool culture EIA for toxin (C diff)
	Viral	*Adenovirus* *Astrovirus* *Cytomegalovirus* *Norovirus* *Rotavirus* *Sapovirus*	Multiplex stool PCR panel Biopsy Pathogen specific PCR (ADV)
	Parasitic	*Cryptosporidium* *Cyclospora* *Entamoeba* *Giardia* *Isospora*	Stool for O&P Multiplex stool PCR panel PCR (giardia)
	Mycobacterial	*Mycobacterium avium* *Mycobacterium gordonae* *Mycobacterium bovis* *Mycobacterium tuberculosis*	Biopsy Interferon-γ release assay
Non-Infectious Causes	Type	Causes	Common diagnostic techniques
	Immunologic	Graft vs. host disease Post-transplant lymphoproliferative disorder Thrombotic microangiopathy	Biopsy
	Medication	Antiarrhythmics Antibiotics Magnesium supplements Mycophenolate Sirolimus Tacrolimus	Reduce or stop medication and assess impact

## Diagnosis

A tiered evaluation of diarrhea in transplant recipients is recommended (1, 3). However, in practice, several tests may be ordered in parallel rather than sequentially. *Clostridium difficile* toxin testing and quantitative serum CMV PCR are in the first tier of testing (1, 3) in addition to stool multiplex PCR which has largely replaced stool bacterial culture, viral culture, and ova and parasite evaluation (1). Stool PCR panels are limited in that susceptibility testing is not available and therefore, in some cases, stool culture may still be necessary. In addition to this initial evaluation, a careful assessment for diarrhea causing medications is essential. See Table 1 for additional recommendations.

If initial evaluation is unrevealing and symptoms persist, a colonoscopy and, if indicated, esophagogastroduodenoscopy should be considered.

## Management

### Supportive care

Key to the treatment of diarrhea by any cause is fluid replacement (4). Age-appropriate oral rehydration fluids are preferred with administration of intravenous fluids when oral intake is inadequate (4, 5). Intravenous fluid resuscitation in the case of severe dehydration or hypovolemic shock should use normal saline or lactated Ringer's solution (4). Attention to immunosuppressant levels during diarrheal illnesses is advised due to increase in calcineurin inhibitor and mTOR inhibitor levels induced by enteritis. The risk of renal toxicity is compounded to the concurrent risk of acute renal injury due to hypovolemia (1).

Any unnecessary medications that could cause diarrhea should be modified, held, or discontinued (1). Adjustments of immunosuppressive medications (1, 3), notably mycophenolate mofetil (6, 7) should be considered when possible. The use of antimotility agents depends on the cause, severity, and patient's age (4, 1). Antimotility agents are discouraged in patients <18 years old and any patient with fever or bloody diarrhea (4). Note that most studies in the use of antimotility agents exclude immunocompromised patients so data specific to transplant recipients is lacking outside of viral enteritis (8, 9). The benefit and risks of probiotics in general and in transplant recipients is controversial (10).

### CMV

CMV is the most common etiology of viral enteritis and colitis in transplant patients (2). Intestinal biopsy is critical in diagnosing gastrointestinal disease caused by CMV as serum CMV levels do not necessarily correlate with gastrointestinal (GI) disease. Ganciclovir and its oral prodrug valganciclovir are first line treatment for tissue invasive CMV infections (11, 12). Initial treatment with IV ganciclovir at 5 mg/kg/dose IV every 12 h is recommended due to risk of poor absorption of oral medication (11). Some experts propose using valganciclovir if oral administration is tolerated and no malabsorption is suspected. Serum ganciclovir level monitoring should be considered in these cases (13).

Unlike treatment of tissue invasive CMV among HIV-infected patients where dose frequency is stepped down following an induction phase, ganciclovir should be dosed every 12 h for the duration of therapy in SOT recipients (11). In HCT patients, generally 2–3 weeks of induction dosing of ganciclovir is utilized before transitioning to a lower maintenance dose, especially if high levels of immune suppression are continuing (14). The standard endpoint for CMV treatment is resolution of clinical symptoms and clearance of viremia (11, 12). Due to the potential lack of association between serum CMV viral load and GI disease, determining when to discontinue therapy can be challenging whether viremia is or is not present initially (12, 15). For SOT recipients, regardless of when the diarrhea resolves, treatment of mild colitis in R+ patients should continue at least 3 weeks with longer treatment ≥6 weeks for moderate to severe colitis or any severity in R− patients (15, 16). Some centers perform repeat endoscopy and continue therapy until endoscopic and histopathologic findings resolve (15). Regardless of duration of treatment, relapse is common (15, 16).

We find it reasonable to transition to valganciclovir in SOT recipients once stool frequency is ≤3/day or consistency is non-liquid to reduce risks associated with central venous catheters. Transition to oral therapy is also acceptable in HCT patients when symptoms have improved if no concomitant gut GVHD is present and serum viral levels are not significantly elevated (14).

### Adenovirus

ADV infection occurs frequently in pediatric transplant patients and as with CMV can range from asymptomatic viremia to severe fatal disseminated infection. In both SOT and HCT recipients, children tend to be affected more frequently and earlier in the post-transplant period than adults. Infection can result from reactivation of prior infection or new acquisition of virus. The GI tract is one of several organ systems that can be affected by adenovirus, as isolated colitis, diarrhea in association with a respiratory infection as in Case 1, or as part of disseminated infection. Hepatitis, encephalitis, hemorrhagic cystitis and lower respiratory tract infection are also possible with ADV in transplant recipients (17). In SOT patients, ADV tends to affect the donor organ.

Over 60 serotypes of adenovirus have been described. These are further classified into species A through G. Specific clinical syndromes can result from varying binding of specific viral serotypes to cellular receptors. In pediatric immunocompetent hosts, adenovirus gastroenteritis is most commonly caused by serotypes 40 and 41 (subgroup F). In immunocompromised hosts the range of viral types affecting the gastrointestinal tract is much broader, including groups A, D and G (17).

Diagnosis can be made by PCR from any of these affected sites (blood, upper or lower respiratory samples, urine, stool). When ADV is found in one site such as blood or nasopharyngeal swab in a transplant recipient, other sites including urine and stool should also be assessed for dissemination. However as asymptomatic and prolonged viral shedding are possible, findings need to be correlated with clinical symptoms. Tissue biopsy may be required to diagnose patients with colitis.

As with other viral infections a decrease in immunosuppression is recommended when possible and, when combined with supportive care, may be enough to improve mild gastroenteritis caused by ADV. No antiviral medication is FDA approved for treatment of adenoviral infection, however cidofovir is commonly utilized for infection in severely immunocompromised patients, despite its potential for nephrotoxicity. Doses of 1 mg/kg three times per week or 5 mg/kg once weekly are commonly utilized. Some data support using lower doses to reduce toxicity in pediatric patients with ADV infection (18).

### Other viruses

Norovirus is a leading cause of gastroenteritis globally and while mild and self-limited in the vast majority of people, can cause significant illness in severely immunocompromised patients including those with primary immunodeficiencies, HIV, and transplant patients (19). Infection can be community or nosocomially acquired, the latter common because of the virus' stability in the environment. Diagnosis is generally made on a multiplex stool PCR panel. Oral immunoglobulin for norovirus has been used for treatment of chronic or severe infection in immunocompromised patients with mixed results. Nitazoxanide, which is FDA approved for Cryptosporidium and *Giardia lamblia* infection, is often utilized for the treatment of transplant and other immunocompromised patients with norovirus gastroenteritis. In a study of 195 adult SOT patients, those who received nitazoxanide reported shorter symptom duration and had lower hospitalization rates (20). A phase II randomized trial of nitazoxanide for treatment of acute and chronic norovirus gastroenteritis in both HCT and SOT patients is almost complete (21).

Many other viruses can also cause diarrheal illness in transplant recipients. While most viral gastroenteritis is mild and self-limited in immunocompetent hosts, these infections can cause significant and prolonged symptomatology in transplant patients and even impact graft survival (2). Rotavirus incidence has decreased dramatically since the implementation of global vaccination strategies in infants. The mainstay of treatment is supportive care and no antiviral therapy is available for most causes of viral diarrhea. Decreased immune suppression when a possibility may help to limit the infection. Prolonged shedding of viruses is common in immunocompromised patients. Cellular therapies are being investigated for a range of viral infections in immunocompromised hosts, including CMV and ADV.

### Enteric Bacteria

#### *Campylobacter* species

*Campylobacter* species are purported to be the most common bacterial cause of diarrhea, particularly in developed countries (22–24), but rarely causes systemic invasive disease (22). However, *Campylobacter* bacteremia occurs more frequently in immunocompromised individuals, including those receiving immunosuppressive medications (23–25). While antimicrobial treatment is usually not necessary for uncomplicated *Campylobacter* enteritis, treatment is recommended in immunocompromised patients regardless of severity of illness (1, 22, 24). Due to increased rates of fluoroquinolone resistance, azithromycin should be the empiric agent of choice to treat *Campylobacter* enteritis (24, 25). Aminoglycosides are recommended for severe *Campylobacter* bacteremia or sepsis (23).

*Campylobacter* can be isolated from stool for up to 7 weeks after the initial illness (22) and can colonize the human gastrointestinal tract (24). Probiotics have been demonstrated to reduce colonization in chickens and may benefit humans colonized with *Campylobacter* (24), but their use is controversial in transplant recipients (10). Colonization can confound the clinical assessment of diarrhea when *Campylobacter* is co-detected with alternative etiologies or in patients at risk for invasive CMV disease.

#### Pathogenic *Escherichia coli*

*Escherichia coli* is a common cause of diarrhea in both immunocompetent and immunocompromised hosts. Enterohemorrhagic *E. coli* (EHEC) is managed differently than the other pathogenic *E. coli*. Antimicrobial therapy, especially beta-lactams, should be avoided in patients with diarrhea due to EHEC (1, 26, 27) as Shiga toxins are released during bacterial cells death (28, 29). These as well as other toxins produced by EHEC are associated with complications such as bloody diarrhea, severe colitis, and hemolytic uremic syndrome (HUS) (26). Maintaining careful fluid and electrolyte balance is important. If HUS develops, the resulting anemia may require blood transfusion, and the damage to the kidneys may require dialysis (26). Various toxin neutralizing agents have been proposed to treat EHEC-related complications, but so far the only agent trialed in humans demonstrated no benefit (26, 27).

The rest of the pathogenic *E. coli* can be treated with antibiotics when significant symptoms are present (1). Targeted antimicrobial therapy is ideal when treating infectious diarrhea; however, reliance on molecular diagnostics results in identification of a bacterial etiology without antimicrobial susceptibility data (1). Azithromycin and ciprofloxacin are effective against pathogenic *E. coli* (1, 4). Both have the potential to increase serum calcineurin inhibitor and mTOR inhibitors level (30, 31).

#### Clostridium difficile

Management of *C difficile* infection (CDI) is fairly well established in adult patients based on guidelines of care (32). The treatment of children and immunocompromised patients can be more complicated especially in the context of persistent, recurrent infections as there are less data supporting newer therapeutics and strategies than in adult immunocompetent hosts. Transplant patients are more susceptible to CDI due not only to their immunosuppressed state but also secondary to frequent antibiotic exposure and overall gut dysbiosis and altered microbiome diversity (33). Proton pump inhibitors are also associated with increased risk of CDI.

Symptoms of CDI can range from asymptomatic colonization to watery diarrhea to fulminant colitis and sepsis. Recurrence is not unusual. Colonization is common in the first 1–2 years of life and therefore testing for CDI in this age group is not recommended.

Diagnosis and treatment of CDI in HCT and SOT patients is similar to management strategies in immunocompetent hosts. Treatment recommendations vary depending on severity of infection and whether it is initial or recurrent. Fidaxomicin and oral vancomycin are considered the first line for treatment of initial infection in adult patients. Most pediatric providers would also consider using oral metronidazole as an acceptable first line treatment option in non-severe infection. Discontinuing antibiotics is an important adjunct to specific treatment (34). A first recurrence is treated with the same or alternate agent from the initial infection. Subsequent recurrences and refractory infection treatments including bezlotoxumab and fecal microbiota transplant are discussed in adult guidelines however data are limited in immunocompromised and pediatric patients (32, 33) and are beyond the scope of this review.

### PTLD

PTLD is more common among SOT recipients compared to HCT recipients (35). Among SOT recipients, the incidence of PTLD ranges from 2% to 30% depending upon the organ type; liver, small bowel, and multivisceral transplants carrying the higher risk (36). GI involvement is frequent due to the large number of resident lymphocytes (37). Clinical manifestations of GI PTLD are varied and can be vague: fever, malaise, abdominal fullness, nausea, vomiting, diarrhea, occult gastrointestinal bleeding, frank hematochezia, intestinal obstruction, and intestinal perforation (37).

First line management of PTLD is reduction of immunosuppression (36). Most experts recommend a 50% reduction in calcineurin inhibitor with discontinuation of azathioprine or MMF (37). GI disease typically also requires treatment with rituximab and/or chemotherapy depending on the histology. Consultation with oncologists is essential. CD20+ unclassifiable histopathology, non-destructive PTLD, polymorphic PTLD, and diffuse large B-cell lymphoma can be treated with rituximab (36, 37). If lesions are progressive, cytotoxic chemotherapy is indicated (36, 37). The most recommended chemotherapeutic regimen is rituximab with cyclophosphamide, doxorubicin, oncovin, and prednisone (R-CHOP) (36, 38). There are trials that support the use of low-dose cyclophosphamide and prednisone to treat rituximab-refractory PTLD (39). In the case of classical Hodgkin lymphoma, reduction of immunosuppression is not validated and alternative chemotherapeutic regimens may be recommended (36, 37). Surgical resection may be required in the case of GI hemorrhage or perforation (36).

### GVHD

Acute GI GVHD is a frequent cause of diarrhea in HCT recipients and causes significant morbidity and even mortality. Diarrhea can be watery but also is sometimes associated with hematochezia and significant blood loss. Other symptoms may include nausea, vomiting and abdominal pain depending on whether the upper or lower GI tract(s) are involved. While acute GVHD occurs within 100 days of transplant, chronic disease occurs later. The GI tract is the second most common cause of GVHD after the skin and gut GVHD incidence in pediatric HCT recipients is approximately 25% (40). Diagnosis requires tissue biopsy after infections and other causes are ruled out. Staging depends on volume of diarrhea and then is combined with presence of skin and/or liver GVHD to determine an overall severity grade. Treatment of gut GVHD, like other forms of GVHD, involves increased immunosuppression with steroids generally being first line (41).

### Less common infectious causes of diarrhea

Many more pathogens, opportunistic or not, cause diarrhea in transplant recipients and can not be covered in this review. We have summarized the treatment recommendations for several less common bacterial and parasitic causes of diarrhea (Table 2).

**Table 2:** Treatment of less common or exposure-specific causes of infectious diarrhea.

Pathogens	Treatment*
Non-typhoidal *Salmonella*	Ciprofloxacin 10 mg/kg/dose BID × 7–14 days May need longer duration if severely immunosuppressed.
*Shigella*	Empiric: Ciprofloxacin 15 mg/kg/dose PO BID × 7–10 days Target therapy as necessary based on susceptibilities.
*Vibrio cholerae*	Doxycycline 4 mg/kg/dose IV or PO × 1 dose
*Yersinia*	Third-generation cephalosporins recommended for immunocompromised. Can also use TMP-SMX, aminoglycosides, fluoroquinolones
*Cryptosporidium*	Paromomycin 10 mg/kg/dose TID × 14–28 days >14 days is often required in solid organ transplant recipients. Or nitazoxanide PO BID × 14 days, dose is age dependent • 1 to <4 years: 100 mg • 4 to <12 years: 200 mg • ≥12 years: 500 mg
*Cyclospora*	Trimethoprim-sulfamethoxazole 5 mg/kg/dose QID × 10 days Followed by indefinite thrice weekly secondary prophylaxis.
*Entamoeba*	Metronidazole 15 mg/kg/dose TID × 10 days + Paromomycin 10 mg/kg/dose PO TID × 7 days
*Giardia*	Metronidazole 5 mg/kg/dose (max. 250 mg) TID × 7 days Nitazoxanide or tinidazole can also be used if age appropriate
*Isospora*	Trimethoprim-sulfamethoxazole 5 mg/kg/dose QID × 10 days Followed by indefinite thrice weekly secondary prophylaxis.

* Many of these infections are self-limited, but treatment is recommended for severe infection or moderately to severely immunocompromised patients.

## Conclusion

Diarrhea is a frequent complication in children following both HCT and SOT and can have multiple etiologies. Significant morbidity is common, though mortality is rare. Molecular diagnostics have become the mainstay of infectious etiologies of gastroenteritis. Further study is needed to develop optimal preventative and treatment strategies, specifically in pediatric transplant recipients.
